# The Relationship between Religious Beliefs and Attitudes towards Public Health Infection Prevention Measures among an Ultra-Orthodox Jewish Population during the COVID-19 Pandemic

**DOI:** 10.3390/ijerph19052988

**Published:** 2022-03-04

**Authors:** Bruria Adini, Yoel Cohen, Ahuva Spitz

**Affiliations:** 1 Department of Emergency and Disaster Management, School of Public Health, Sackler Faculty of Medicine, Tel Aviv University, Tel Aviv 6139001, Israel; 2 Moskowitz School of Communication, Ariel University, Ariel 4077625, Israel; prof.yoelcohen@gmail.com; 3 School of Nursing, Jerusalem College of Technology, Jerusalem 91160, Israel; ahuvas@jct.ac.il

**Keywords:** ultra-Orthodox, perceived anxiety, information, COVID-19, public health

## Abstract

The ultra-Orthodox population in Israel was heavily impacted by COVID-19; it is important to understand the factors that contributed to this. There may be a friction between religious versus governmental guidelines that may reduce adherence to COVID mitigation guidelines, such as social distancing and masking. The purpose of this study is to explore this tension and the extent to which it existed in the surveyed sample. The study identified attitudes of ultra-Orthodox individuals concerning religious and public health measures to mitigate COVID-19 infection. A closed-ended questionnaire was completed by 405 ultra-Orthodox Jews. Most respondents believe that religious learning protects from harm (91%); 74% believe that periodically there are inconsistencies between religious guidelines and medical guidelines; 59% believe that preventive medicine may clash with “Divine protection”. Some public health measures applied to contain the pandemic threaten religious lifestyle; this is a source of dissonance among ultra-religious populations, which may substantially decrease willingness to comply with public health measures.

## 1. Introduction

Flattening the curve of the COVID-19 pandemic and keeping it from rising again necessitate active involvement and compliance of the population with the policies of the government [1]. Individuals or groups within societies react differently to the instructions relayed by the authorities, most especially minority groups [2,3]. The extent to which guidelines and regulations are understood the degree of trust in authorities, risk perceptions, exposure to the media, and cultural diversities are just some of the factors that may be related to the willingness or lack thereof of groups to adhere to the regulations [4,5]. Since these elements may be perceived differently, it is important to identify the specific beliefs, attitudes and perceptions of different societal groups. This article focuses upon the ultra-Orthodox or “Haredi” Jewish population in Israel, which maintains a distinctly religious-oriented identity [6,7].

The prevalence of COVID-19 infection in Israel varied among the different cultural and ethnic groups. At the initial phases of the pandemic, in March 2020, more than 40% of confirmed cases were identified among the ultra-Orthodox Jews, even though their representation in the society is only 12.5% [6,8,9]. Some of the characteristics of this specific sector of the population may have contributed to these high rates of infection, notably the dense populations, crowded housing conditions, a low level of trust in authorities, a high dependency on rabbinical (religious) rulings, a lower socio-economic level, as well as limited access to data that are generated by “secular” media sources [8,10]. Attitudes and perceptions of ultra-Orthodox Jews are often derived from religious beliefs, consequently impacting upon behaviors and compliance with rules and regulations [10,11].

During the COVID-19 pandemic, the ultra-Orthodox Haredi lifestyle resulted in tension between instructions relayed by their rabbinical leaders and the instructions and advisories from the public health authorities [6,10]. For example, studying the Talmud in the framework of partners learning together, for many hours, is considered a virtue, but it was strongly discouraged by the health authorities. Also discouraged was communal prayer in the synagogue—advising individual prayer at home. Such close-knit gatherings were prohibited during the pandemic, due to the need to maintain social distancing. Issuing lockdowns and curfews (a measure adopted by many countries) [12,13] during holy religious holidays was perceived by ultra-Orthodox individuals as a threat to their core values and strongly felt beliefs [14]. While efforts were made to try to bridge gaps between religious beliefs and public health guidelines, the ultra-Orthodox community frequently perceived that it was being discriminated against and stigmatized [6,15]. 

Attitudes and perceptions are based to a large extent on exposure to relevant information [16]. While the search for information in varied media channels frequently rises during times of crisis, ultra-Orthodox Jews may refrain from such open search for information [17]. The ultra-Orthodox population is characterized by limited access to data, as it mainly utilizes communication channels that reflect their own lifestyle—notable modesty as a benchmark—and even more so, to information relayed directly by their religious leaders, because communication with them is regarded as highly desirable and effective [15]. To varying degrees, some Haredi rabbis have imposed over the years limitations, or filters, on the Internet, arguing that undesirable content might damage the religious Jewish home [18].

During the COVID-19 pandemic, all this caused ultra-Orthodox Haredi individuals to be exposed to only part of the information available to the broad population, and official guidelines by health authorities were subject to misinterpretation [7,15]. With rabbinical leaders an important source of information, instructing their followers to continue to practice their religious customs, mistrust of information disseminated by secular channels of communication grew, even when the source was governmental. [6]. Furthermore, with regards to collective worship, many Haredi men feel purposeless when they cannot pray together. This led to a conflict between compliance with governmental guidelines concerning COVID-19 versus adherence to those given by the rabbinical leaders [15]. It should be noted that gender differences [19,20] may impact on the attitudes and beliefs as a result of the varied exposure to the religious leadership; while men are required to regularly pray three times a day, in the synagogue if possible, women are not expected to do so [14,21]. 

The aims of the study were to identify (1) the communication means used to access information concerning the pandemic; and (2) beliefs of ultra-Orthodox Jews concerning the intersection between religion and public health measures to prevent COVID-19 infection. 

## 2. Materials and Methods

### 2.1. Study Population

The city of Bnei Beraq, which has a population of 209,651 residents (according to the Israeli Central Bureau of Statistics), consists solely of ultra-Orthodox individuals, and thus, was chosen for the study. The required sample size was calculated using the OpenEpi online calculator [22]. Based on the size of the ultra-Orthodox population in Israel, which consists of 1,175,000 people, with confidence limits of 5% and hypothesized 50% ± 5 frequency of outcome factor in the population, 385 participants were required. The interviewing researchers were instructed to include only respondents that were 18 years or older, of both genders. A sample of the ultra-Orthodox Haredi population (*N* = 405) was produced with the objective of identifying trends in the information flow concerning religious and health measures during COVID-19.

### 2.2. Study Design

The study was a cross-sectional design, conducted during October 2020, at the peak of the second wave of COVID-19. Given that effective management of the COVID-19 pandemic is dependent on the cooperation of all sectors of the population, and as the ultra-Orthodox Haredi population constitutes 12.5% of the Israeli populace [7,9], a quantitative study was conducted. Considering the unique characteristics of the ultra-Orthodox population (low access to internet; limited contact with non-Orthodox individuals; relatively low socio-economic levels, among other factors), participants for the study were recruited by two religious researchers, who approached the respondents through a telephone call, explaining that the study was being done for the purpose of understanding the distinctive perceptions of the ultra-Orthodox population. The respondents were chosen randomly, based on the land-line phone directory, reaching out to every eighth name, according to the alphabetical order. After four failed attempts to reach a specific person, the researchers telephoned the individual who was listed next in the directory. The overall dropout rate (people who refused to partake in the study) was less than 25%. After obtaining informed participant consent, a structured questionnaire was filled in by the researchers. All responses were recorded anonymously by the researchers in Excel spreadsheets, and then transferred to SPSS software version 26. The study was approved by the Ethics Committee of Tel Aviv University, number 0001312-1, dated 14 April 2020.

### 2.3. Study Tool

The survey was done based on a structured closed-ended questionnaire that was developed specifically for the study. The study included the following main components: 1. Attitudes and beliefs concerning religious and public health measures to mitigate COVID-19 infection (10 items); 2. Communication channels used to access information on COVID-19 (6 items); 3. Factors that motivate search for information on COVID-19 (4 items); 4. level of trust in the information relayed (7 items); 5. Demographic data including gender, age, level of income, time exposed to the media, and the pre-COVID-19 degree of communication with the rabbi, ranging from “not good at all” to “very good”. Except for the communication, exposure to information and demographic data, all variables were measured based on a five-point Likert scale, with 1 being to a very low extent and 5 being to a very high extent. 

### 2.4. Analysis of Data

Descriptive statistics were used to analyze the beliefs and perceptions of the subjects in the sample. One-way analysis of variance and Chi Square tests were used to identify significance of differences between numerical and nominal variables, respectively. All statistical analyses were performed using SPSS software version 26. *p*-values lower than 0.05 were considered to be statistically significant.

## 3. Results

The number of respondents who answered the questionnaire was 405. Among them 49% were females; the mean age was 44 years, ranging from 19 years to 88 years. A third of the population had less than 12 years of education, and the majority (74%) had an income that is much lower than the average salary in Israel. The mean number of children was 5 ranging from none to 12 children. The respondents belonged to various sections of the ultra-Orthodox population. See Table 1. 

### 3.1. Beliefs and Perceptions Concerning Religion and Public Health Measures to Mitigate COVID-19 Infection among the Ultra-Orthodox Population

**Figure 1:**
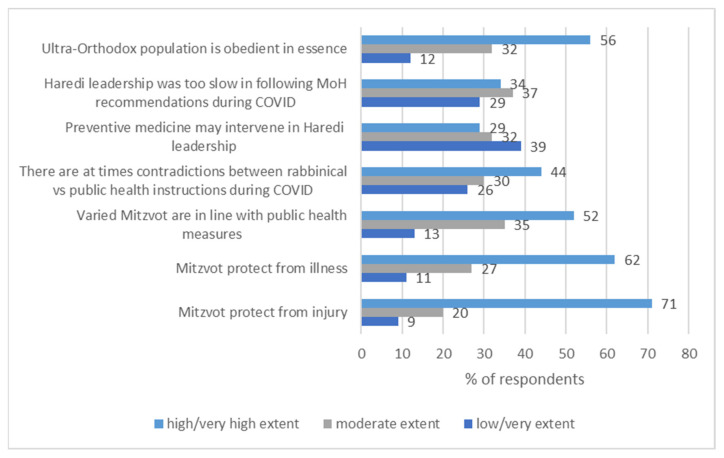
Beliefs and perceptions of ultra-Orthodox respondents concerning religion and public health measures to mitigate COVID-19 infection, in percentages (*N* = 405).

Most of the ultra-Orthodox respondents (91%) believe (from “moderate degree” to a “very high degree”) that religious learning and observance of the religious commandments (“mitzvot”) protect from bodily harm (91%) and against illness (89%). The majority perceive that that the varied “Mitzvot” are in line with medical recommendations (87%). However, in the case of COVID-19, 74% believe (from “moderately” to “very high extent”) that periodically there are contradictions between the two. Somewhat contradictorily, while 61% believe (“moderately” to a “very high extent”) that preventive medicine may be interpreted as intervening with the ‘Divine protection’, or is excessive, 71% thought that the rabbinical leadership was too slow in adopting the recommendations of the medical experts in relation to COVID-19. Most respondents (88%) perceive (from “moderate degree” to “very high degree”) that the ultra-Orthodox population does, in essence, adhere to the regulations of the health authorities. See Figure 1.

**Figure 2:**
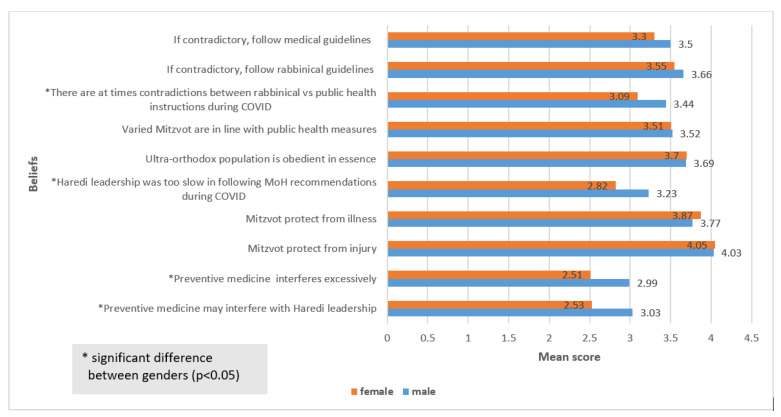
Mean scores of beliefs of males and females concerning religious versus public health measures to mitigate COVID-19 infection (*N* = 405), on a scale of 1 (do not believe at all) to 5 (fully believe).

Some differences were found between males and females regarding the beliefs and perceptions, most especially in regard to the relationship between religious versus public health measures to mitigate COVID-19 infection. The comparison between males and females is presented in Figure 2.

No significant differences were found with regard to beliefs and perceptions concerning religious versus public health measures to mitigate COVID-19 infection among the ultra-Orthodox population, according to their age or respective levels of income.

### 3.2. Communication Channels Used to Access Information Concerning COVID-19

**Figure 3:**
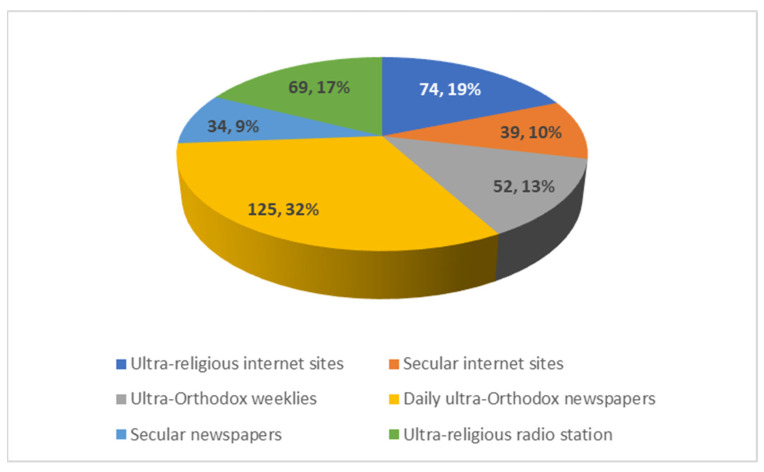
Number and percentage of communication types used to access information on COVID-19 (*N* = 393).

The respondents utilized varied communication channels to access information concerning COVID-19. The respondents were asked to mark each channel that they used to access such information. See Figure 3. 

The factors that motivated the respondents to look for information concerning COVID-19, in a “moderate degree” to “very high degree”, included finding rabbinical rulings (Halakha) (54% of respondents), articles about rabbis (55% of respondents), information about individuals that died as a result of COVID-19 (67% of respondents), or news concerning old-age homes (58% of respondents). 

### 3.3. Beliefs in the Information Concerning COVID-19 and Its Adaptability to the Ultra-Orthodox Lifestyle

The extent to which respondents believed the information relayed by the media was relatively low, though a higher percentage of respondents highly believed in the information relayed by the ultra-Orthodox media (38%) compared to the secular media (27%). Only a little more than a third of the sample population (38%) feel that the information concerning COVID-19 was highly or very highly in line with their ultra-Orthodox lifestyle, while 62% feel that it is adaptable to their life style only to a very small to moderate degree. 

**Figure 4:**
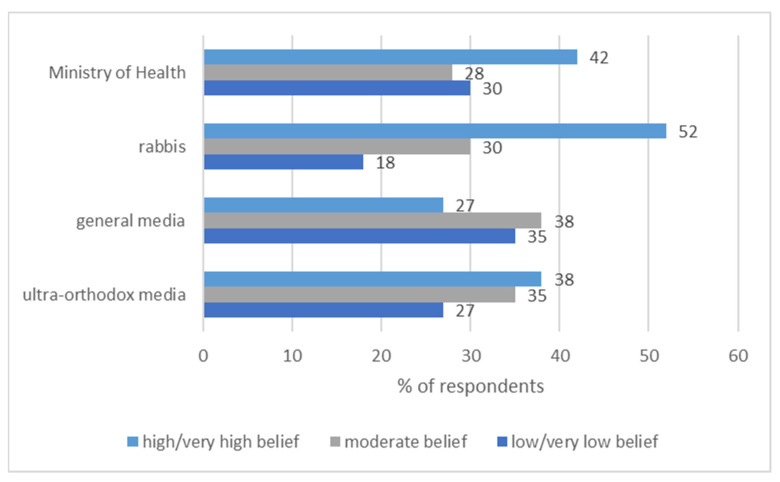
Belief in information according to its primary source by percentage (*N* = 405).

When comparing the level of trust in the information, substantial differences were found according to its primary source (either from religious or from medical leaders). Fifty-two percent of the respondents highly or very highly believe in information concerning COVID-19 provided by their religious leaders (rabbis) compared to only 42% that trust the information disseminated by the Ministry of Health. See Figure 4.

When there are discrepancies between the instructions given by rabbinical leaders and those of medical providers, 56% believe to a “high extent” or “very high extent” that the right course is to act according to the guidelines issued by their religious leaders, while 27% and 18% believe that this is the right course of action to a “moderate extent” or “small extent”, respectively. 

## 4. Discussion

Managing the COVID-19 pandemic is a global challenge, which necessitates the active and effective integration of civil societies, in response to the varied encountered complexities [1]. It is vital to understand the diverse cultural, social, economic, and normative contexts of the different populations and their specific needs and expectations in order to increase their adherence to the mitigating guidelines and regulations [14,15]. A specific minority group of ultra-Orthodox Jews was investigated during the COVID-19 pandemic, to identify the communication means they use to access information and facilitate the understanding of their public health recommendations for infection prevention versus religious beliefs concerning the COVID-19 pandemic. 

Consistent with previous studies, a high majority of the interviewed ultra-Orthodox Jews were found to believe that religious studies and observance of the religious laws protect against illness [10,23]. Considering that public health measures applied to contain the pandemic—especially social distancing and lockdowns—threaten religious observance (including prayer meetings or group religious study), this is a potential source of dissonance among the ultra-Orthodox population [6,24]. This concern is reflected by the finding that 74% of the ultra-Orthodox respondents believe that there are at times contradictions between the different religious observances and medical recommendations issued during the COVID-19 pandemic. Considering that the ultra-Orthodox Haredi population is in its essence an “obedient” group [15], and mostly follow the decrees issued by their rabbinical leaders [25], this substantially decreases their willingness to comply with measures that are perceived as not being in line with their religious lifestyle [6,26].

In line with previous findings [27,28], significant differences were found among males and females with regard to discrepancies between public health measures and religious observances. This was especially discerned in this study concerning the belief in preventive medicine measures. Men were found to perceive these measures as being excessive or interfering with their religious rituals to a higher extent than women. These may have resulted from the gender-specific responsibilities that are characteristic of the ultra-Orthodox society, whereby many men focus mainly on religious studies and, thus, have less interactions with the wider eco-systems, while women are the main providers for the families, and more closely interact with medical sources and health authorities [29]. The women, therefore, may be more capable of balancing the two “worlds”, while the men find it more difficult to adapt to the new reality that is enforced, not allowing them to continue their pre-COVID routines, particularly concerning joint praying or learning activities [25,27]. In contrast, women perceived to a lesser degree than men that at times there may be contradictions between the rabbinical versus the public health recommendations for infection prevention or that the Haredi rabbis were too slow in following those recommendations of the medical experts. This difference too is most probably influenced by the capacity of women to better maneuver between the Haredi life and the characteristics of the overall society [27,29]. 

Governmental guidelines on behavior during COVID-19 were mainly disseminated through the varied media and communication channels. The ultra-Orthodox population is characterized by a limited exposure to information as well as with a low level of trust in non-Haredi authorities and media sources [27,30]. Accordingly, this may lead to reluctance to adhere to the instructions relayed through these media channels [31], even more so because many of the ultra-Orthodox media filter the information that is relayed by governmental authorities and focus only on those guidelines issued by the rabbinical leadership, compromising the level of compliance of the ultra-Orthodox population [7]. This problem may intensify when the ultra-Orthodox population perceives an inconsistency between medical versus religious leadership or when there is a disruption of communication with their religious leaders, who serve as a source of information and guidance [15,18]. Furthermore, this dissonance may lead to varied ‘conspiracy theories’ [32] and beliefs that the ultra-Orthodox population is being discriminated against [30]. This breakdown in communication should be further explored.

### Limitations

Four main limitations should be considered. The first is that the study was conducted using the telephone directory of Bnei Beraq, which may lack information concerning people who do not have landline phones. Furthermore, as the ultra-Orthodox population is known to be a very close-knit community, it should be taken into account that they may not feel completely comfortable sharing their perceptions concerning controversial issues, such as inconsistencies between religious versus public health guidelines. Despite the value of focusing on the single all-Haredi city of Bnei Beraq, future research could benefit from looking at other types of ultra-Orthodox populations who may live under different conditions. The study was based on a questionnaire, which has characteristic limitations. 

## 5. Conclusions

Understanding the unique characteristics of minority populations is vital for authorities and emergency managers, in order to effectively integrate them in the response and mitigation plans and policies. As the ultra-Orthodox population is one such group, a study was conducted to identify their ways of accessing information during the COVID-19 pandemic and their distinctive beliefs and attitudes concerning potential discrepancies between religious and public health measures to mitigate COVID-19 infection. The important insights that were discovered, including inconsistencies between the guidelines relayed by religious leaders versus the health authorities as well as gender diversities, should lay a foundation to a more effective collaboration between authorities and this specific population. It is recommended that additional studies be conducted in varied types of religious communities and contextual conditions, to investigate whether or not these findings also characterize their populaces. 

## Figures and Tables

**Table 1:** Characteristics of study population (*N* = 405).

Variable	Group	Number & Standard Deviation (SD)	Percentage
Mean age		43.9 (13.57)	
Gender	Males	202	51%
	Females	195	49%
Level of education			
	<12 years	141	34.8%
	Seminary	42	10.4%
	Yeshiva	140	34.6%
	Academic	82	20.2%
Mean number of children		5 (2.4)	
Level of income (in US$)	Less than 3100	299	73.8%
	>3100	86	21.2%
Sub-groups of the ultra-Orthodox population	Ashkenazi	73	18%
	Hassidic	141	35%
	Haredi (Lithuanian or Non-Hassidic)	125	32%
	Sepharadic	52	13%
	other	14	3%

## Data Availability

The analyzed data are available through the authors. The raw data are not publicly accessible due to ethical constraints.
